# Metformin Induces Apoptosis and Cell Cycle Arrest Mediated by Oxidative Stress, AMPK and FOXO3a in MCF-7 Breast Cancer Cells

**DOI:** 10.1371/journal.pone.0098207

**Published:** 2014-05-23

**Authors:** Eveline A. I. F. Queiroz, Stephanie Puukila, Rosangela Eichler, Sandra C. Sampaio, Heidi L. Forsyth, Simon J. Lees, Aneli M. Barbosa, Robert F. H. Dekker, Zuleica B. Fortes, Neelam Khaper

**Affiliations:** 1 Pharmacology Department, Institute of Biomedical Sciences, University of São Paulo, São Paulo, São Paulo, Brazil; 2 Department of Physiological Sciences, State University of Londrina, Londrina, Paraná, Brazil; 3 Biorefining Research Institute, Lakehead University, Thunder Bay, Ontario, Canada; 4 Northern Ontario School of Medicine, Lakehead University, Thunder Bay, Ontario, Canada; 5 Biology Department, Lakehead University, Thunder Bay, Ontario, Canada; National Cancer Center, Japan

## Abstract

Recent studies have demonstrated that the anti-diabetic drug, metformin, can exhibit direct antitumoral effects, or can indirectly decrease tumor proliferation by improving insulin sensitivity. Despite these recent advances, the underlying molecular mechanisms involved in decreasing tumor formation are not well understood. In this study, we examined the antiproliferative role and mechanism of action of metformin in MCF-7 cancer cells treated with 10 mM of metformin for 24, 48, and 72 hours. Using BrdU and the MTT assay, it was found that metformin demonstrated an antiproliferative effect in MCF-7 cells that occurred in a time- and concentration- dependent manner. Flow cytometry was used to analyze markers of cell cycle, apoptosis, necrosis and oxidative stress. Exposure to metformin induced cell cycle arrest in G_0_-G_1_ phase and increased cell apoptosis and necrosis, which were associated with increased oxidative stress. Gene and protein expression were determined in MCF-7 cells by real time RT-PCR and western blotting, respectively. In MCF-7 cells metformin decreased the activation of IRβ, Akt and ERK1/2, increased p-AMPK, FOXO3a, p27, Bax and cleaved caspase-3, and decreased phosphorylation of p70S6K and Bcl-2 protein expression. Co-treatment with metformin and H_2_O_2_ increased oxidative stress which was associated with reduced cell number. In the presence of metformin, treating with SOD and catalase improved cell viability. Treatment with metformin resulted in an increase in p-p38 MAPK, catalase, MnSOD and Cu/Zn SOD protein expression. These results show that metformin has an antiproliferative effect associated with cell cycle arrest and apoptosis, which is mediated by oxidative stress, as well as AMPK and FOXO3a activation. Our study further reinforces the potential benefit of metformin in cancer treatment and provides novel mechanistic insight into its antiproliferative role.

## Introduction

The prevalence of cancer, a multi-factorial disease, is increasing at an alarming rate worldwide. According to GLOBOCAN, breast cancer is now the most common cancer both in developed and developing regions, with an estimated 1.38 million new cancer cases diagnosed in 2008 [1], [2]. Due to rising numbers of new cancer cases, developing and discovering treatment for cancer that minimizes side effects is of utmost priority.

Metformin is an anti-diabetic drug that is commonly prescribed to treat type 2 diabetes and has recently received attention as a potentially useful therapeutic agent for treating cancer [3]–[6]. Metformin lowers elevated insulin levels associated with type 2 diabetes by inhibiting hepatic gluconeogenesis via AMP-activated protein kinase (AMPK) activation. It increases insulin sensitivity and glucose utilization by skeletal muscle and adipose tissue resulting in reduced blood glucose and insulin levels [7], [8]. Metformin can have a direct antitumoral effect, but also can act indirectly to improve insulin sensitivity, decrease hyperinsulinaemia and consequently decrease tumor proliferation [9]–[11]. The decrease in insulin levels caused by metformin can reduce the activation of insulin pathways such as PI3K/Akt/mTOR and MEK/ERK1/2 and lead to a decrease in tumor growth [10]. Akt regulates cell cycle and proliferation directly by targeting p27 and indirectly by modulating levels of cyclin D1 [12]. Metformin can activate the LKB1/AMPK pathway and inhibit cancer cell growth by inhibiting mTOR activity [13]–[16]. This energy sensing LKB1/AMPK pathway regulates phosphorylation of p27 by mediating either cell survival or apoptosis [17]. Furthermore, studies have shown that AMPK can activate the forkhead transcription factor (FOXO) proteins under certain conditions, such as nutrient deprivation leading to increased cell survival [18], [19].

FOXO proteins (FOXO1, FOXO3a, FOXO4 and FOXO6) are an evolutionarily conserved subfamily of transcription factors involved in a variety of cellular processes including tumor suppression [20], [21]. These proteins can induce tumor suppression by promoting cell cycle arrest, repairing damaged DNA and causing apoptosis by up-regulating specific gene expression [12], [22], [23].

While some therapeutic agents used to treat cancer involve mechanisms that directly target apoptosis in tumor cells, most of the therapeutic agents interfere with DNA replication and can affect progression through the cell cycle. Cell cycle progression is altered in a variety of tumors and is often due to mutations or over-expression of genes that code for proteins involved in regulating cell cycle, such as cyclin D1 and p27. Cell cycle arrest in the Sub G_1_, G_0_ and G_1_ phases can lead to apoptosis [24]. While oxidative stress has been linked to cancer, antioxidants have been reported to reduce the risk of certain cancers [25].

The objectives of the present study were to investigate the antiproliferative role of metformin in MCF-7 breast cancer cells, and elucidate the role of FOXO3a and AMPK activities in these cells. In order to achieve this, MCF-7 cells were incubated with 10 mM metformin and markers of oxidative stress, apoptosis, necrosis and cell cycle were analyzed by flow cytometry. In order to elucidate the downstream signaling pathway involved in apoptosis and insulin signaling, western blot and real time RT-PCR were performed. The expression of Bax, Bcl-2, caspase-3 and -7, as well as cleaved caspase-3 were studied as indicators of apoptosis. To further confirm the involvement of oxidative stress and better understand its role in apoptosis, MCF-7 cells were also treated with a variety of antioxidant enzymes apocynin, superoxide dismutase (SOD), catalase, manganese (Mn) SOD, and copper/zinc (Cu/Zn) SOD.

## Materials and Methods

### Cell line and culture conditions

Breast cancer MCF-7 cells (American Type Culture Collection) were cultured in Dulbecco's Modified Eagle Medium (DMEM, Sigma-Aldrich, Oakville, ON, Canada) containing 25 mM glucose, supplemented with 10% fetal bovine serum (FBS) and antibiotic/antimycotic (100 units mL^−1^ penicillin G sodium, 100 µg mL^−1^ streptomycin sulfate, 0.25 µg mL^−1^ amphotericin B). Cell cultures were maintained in T-75 flasks, in a humidified atmosphere at 37°C and 5% CO_2_. Cell culture medium was changed every four days and cells were sub-cultured upon reaching 85% confluence.

The LLC WRC-256 rat Walker tumor cell line is a carcinoma cell line established by Hull [26]. The LLC WRC-256 tumor cells were cultured in minimum essential medium (MEM) supplemented with 10% fetal calf serum (FCS) and antibiotics (50 units mL^−1^ penicillin G sodium and 50 µg mL^−1^ streptomycin sulfate) in a humidified atmosphere at 37°C and 5% CO_2_. Cell culture medium was changed every four days and cells were sub-cultured upon reaching 85% confluence.

### In vitro cell proliferation (MTT) assay

The metabolic activity of living cells, an indication of proliferation and viability, was determined by using the 3-(4,5-dimethylthiazol-2-yl)-2,5-diphenyltetrazolium bromide (MTT) assay. MCF-7 and LLC WRC-256 cells (150 µL; 2.5×10^4^ cells per well) were cultured in 96-well microplates and incubated in medium containing 10% FBS or FCS. After incubation for 24 hours, MCF-7 cells were treated with a series of concentrations of metformin (2.5, 5, 10 and 20 mM, Sigma-Aldrich, Oakville, ON, Canada), as used in previous studies [14]–[16]. After the cells were treated with metformin, they were incubated for a further 24, 48 and 72 hours at 37°C and 5% CO_2_. After incubation for 24 hours, LCC WRC-256 cells were treated with a series of concentrations of metformin (0.5, 1.25, 2.5, and 5 mM) and incubated for 24 or 48 hours at 37°C and 5% CO_2_. For experiments involving oxidative stress, the cells were incubated with 1 mM of hydrogen peroxide (H_2_O_2_) for 4 hours. After this period, reduction of MTT was determined following manufacturer's instructions (Sigma-Aldrich, Oakville, ON, Canada). The treatment groups were compared with the control group and the results were expressed as number of viable cells.

### BrdU Incorporation

After 48 hours of treatment with 10 mM metformin, cell proliferation was measured via 5-bromo-2′-deoxyuridine (BrdU) incorporation as described by Lees et al. [27]. Briefly, MCF-7 cells were treated with 10 µM BrdU for 30 minutes at 37°C and 5% CO_2_. Subsequently, the cells were washed with phosphate buffered saline (PBS), trypsinized, and quenched with media. After centrifugation, cells were resuspended in PBS and fixed with ice cold 70% ethanol. Fixed cells were stored at 4°C until further analysis.

For detection of BrdU incorporation, cells were incubated at room temperature in 6 M hydrochloric acid for 30 minutes. Afterwards, the cells were washed twice with 0.1 M borate buffer to neutralize the acid and then washed once with PBS containing 0.1% BSA. Cells were then incubated with anti-BrdU fluorescein (10 µg mL^−1^) for 30 minutes at room temperature in the dark. Twenty-thousand cells were analyzed using flow cytometry and the CellQuest Pro software.

### Cell cycle analysis by flow cytometry

MCF-7 cells (5.0×10^5^ cells per well) were cultured in 6 well/plates and allowed to adhere to the well walls overnight. After incubation for 24 hours, the cells were treated with 10 mM of metformin for 24, 48 and 72 hours at 37°C and 5% CO_2_. Subsequently, the cells were collected, washed in PBS and fixed in 70% ethanol at 4°C overnight. Then, the cells were centrifuged at 380×*g* for 5 minutes at room temperature. The supernatant (ethanol) was removed and the cells were resuspended in 4 mL of PBS and centrifuged again. The supernatant was removed and the cell pellet was resuspended in 400 µL of PBS. The cell solution was transferred to a flow tube, 10 µL of RNase A (10 mg mL^−1^) was added and the samples were incubated for 1 hour at 37°C. Finally, 64 µL of PI (propidium iodide, 250 µg mL^−1^) was added and the cells were incubated for an additional 15 minutes in the dark at 37°C. The cell cycle distribution was determined by flow cytometry for DNA content (FACScan, Becton Dickinson, Germany).

Four cursors were added in a horizontal position to quantify the different phases (Sub G_1_, G_0_-G_1_, S and G2-M) of cell cycle. Results were presented as percentage of MCF-7 cells in each phase of cell cycle in relation to total number of cells counted.

### CaspaTag caspase-3/7 in situ assay

Active caspase-3/7 positive cells were assessed by flow cytometry according to the manufacturer's protocol (Chemicon, Temecula, USA). MCF-7 cells were treated with metformin (10 mM for 48 and 72 hours) then stained with Fluorochrome Inhibitors of Caspases (FLICA) and incubated for 1 hour at 37°C. Cells were mixed every 20 minutes during staining. FLICA was then aspirated and the cells were washed with 1X wash buffer. Active caspase-3/7 positive cells were assessed by flow cytometry based on their green fluorescence following incubation with carboxyfluorescein-labeled fluromethyl ketone peptide inhibitor of caspase-3. The activation of caspase-3/7 was expressed as the mean fluorescence.

### Necrosis assay

A suspension of MCF-7 cells (6.0×10^5^ cells/well; 3 mL) was seeded onto 6 well/plate and allowed to adhere to the wall overnight. After 24 hours, an equal volume (3 mL) of metformin solution (10 mM) was added to each well, and the cells were incubated for 48 and 72 hours at 37°C and 5% CO_2_. Then, the cells were removed from the plates and centrifuged at 380×*g* for 5 minutes at room temperature. The supernatant was removed, and the cells washed twice with 1X wash buffer. Finally, the cell pellet was resuspended in 400 µL of 1X wash buffer and transferred to a flow tube, followed by the addition of 2 µL of PI solution (250 µg mL^−1^). The percentage of PI positive cells was determined using flow cytometry.

### Apoptosis assay

Apoptosis was assessed via flow cytometric analysis of control and metformin treated cells that were stained with FITC-Annexin V and PI using the Annexin V-FITC Apoptosis Detection kit according to the manufacturer's protocol (Sigma-Aldrich, Oakville, ON, Canada). MCF-7 cells (1.0×10^6^ cells per flask) were seeded onto sterile flat-bottom 25 cm^2^ culture flasks and allowed to adhere to the flask walls overnight. After incubation for 24 hours, the cells were treated with 10 mM of metformin for 24 or 48 hours at 37°C and 5% CO_2_. One flask was treated with staurosporine (2 µg mL^−1^) for 2 hours to induce apoptosis. Subsequently, the cells were collected, washed in PBS and resuspended in 500 µL of 1X annexin-binding buffer at 1.0×10^6^ cells mL^−1^. Cells were then incubated at room temperature with annexin V-FITC and PI stain in the absence of light. Following the 10 minute incubation, samples were immediately analyzed via flow cytometry. Annexin V staining was detected as green fluorescence and PI as red fluorescence.

### Western blotting

The MCF-7 cells were treated with 10 mM metformin for 24, 48 and 72 hours. After which, the cells were homogenized in 200 µL of lysis buffer. Cell debris was removed by centrifugation at 12,000×*g* for 30 minutes at 4°C, and the protein content determined using a Bradford assay (Bio-Rad, Mississauga, ON, Canada). From each sample, 50 µg of protein was boiled and subjected to electrophoresis in denaturing 10% SDS-PAGE. Proteins were transferred to polyvinylidene fluoride (PVDF) membranes using a Trans-blot apparatus (Bio-Rad, Mississauga, ON, Canada). The membranes were blocked with 5% BSA (bovine serum albumin) for 1.5 hours at room temperature. The membrane was then incubated with the appropriate antibody overnight at 4°C. Following washing in 1X Tris buffered saline (TBS; pH 7.4), the membrane was incubated with the appropriate horseradish peroxidase (HRP)-conjugated secondary antibody for 2 hours at room temperature. Finally, the membrane was washed in 1X TBS, and protein expression was visualized using an enhanced chemiluminescence reagent. All primary antibodies were purchased from Cell Signaling Technologies (Danvers, MA, USA), except for Bax and Bcl-2 antibodies which were purchased from Santa Cruz Biotechnology (Santa Cruz, CA, USA). The dilutions of the antibodies were as follows: anti-IR (1∶100), anti-p-IR (Tyr1162/1163) (1∶100), anti-Akt (1∶1000), anti-p-Akt (Ser413) (1∶1000), anti-ERK1/2 (1∶1000), anti-p-ERK1/2 (Thr202/Tyr204) (1∶1000), anti-p38 (1∶1000), anti-p-p38 (Thr180/Tyr182) (1∶1000), anti-AMPKα (1∶1000), anti-p-AMPKα (Thr172) (1∶500), anti-p70S6K (1∶1000), anti-p-p70S6K (Thr389) (1∶1000), anti-FOXO3a (1∶1000), anti-p-FOXO3a (Ser253) (1∶200), anti-p-FOXO3a (Ser413) (1∶500), anti-p27 (1∶1000), anti-Bax (1∶100), anti-Bcl-2 (1∶100), anti-cleaved caspase-3 (1∶1000), anti-MnSOD (1∶2000), anti- Cu/ZnSOD (1∶1000), anti-catalase (1∶3000) and anti-β-actin (1∶5000). The secondary antibody, anti-rabbit IgG (1∶5000; Cell Signaling Technologies), was used for all primary antibodies, except for Bcl-2, catalase and β-actin which used anti-mouse IgG (1∶2000, 1∶5000 and 1∶5000, respectively) and for p-IR which used anti-goat IgG (1∶2000). The dilutions were made in 5% BSA solution.

All blots represent at least 3 different samples for each group. All quantification was normalized to the β-actin level. Data were expressed as mean ±SEM of the ratio between the signal for the protein of interest and the β-actin signal using the Image J program. Phosphorylated protein was compared with total protein and expressed as the ratio between phosphorylated and total protein.

### Determination of mRNA level

MCF-7 cells were treated with 10 mM metformin for 24, 48 and 72 hours. LLC WRC-256 cells were treated with 5 mM metformin for 48 hours. Total RNA samples were prepared using the Aurum total RNA mini kit (Bio-Rad, Mississauga, ON, Canada) according to the manufacturer's protocol, and the integrity of RNA was analyzed by the Experion RNA StdSens Analysis Kit (Bio-Rad, Mississauga, ON, Canada). Following this step, cDNA was prepared using the Fermentas cDNA kit (Fermentas, Burlington, ON, Canada), and the mRNA level was analyzed by real time PCR using iCycler iQ5 (Bio-Rad, Mississauga, ON, Canada) and the iCycler optical system software (version 3.1) using SYBR Green PCR Master Mix. The results were normalized to GAPDH and expressed relative to the control condition.

### CM-H_2_DCFDA assay

A suspension of MCF-7 cells (1.0×10^6^ cells/flask) was cultured in T-25 flasks and allowed to adhere to the wall overnight at 37°C and 5% CO_2_. After 24 hours, the bound cells were treated with the treatment solution (10 mM metformin) for 48 and 72 hours at 37°C and 5% CO_2_. The medium was removed, and the cells were incubated with 1 mL of CM-H_2_DCFDA (5-(and-6)-chloromethyl-2′,7′-dichlorodihydrofluorescein diacetate acetyl ester) solution for 30 minutes. After this incubation period, the dye solution was removed by aspiration, and the cells were trypsinized and centrifuged at 500×*g* for 5 minutes at 4°C. Finally, the cells were washed twice with PBS, left on ice and kept in the dark. The samples were analyzed by flow cytometry. To analyze the quantity of reactive oxygen species (ROS) produced in the MCF-7 cells, we measured the fluorescence of CM-H_2_DCFDA at 488 nm on the FL1 channel of a BD FACSCalibur Flow Cytometer (BD Biosciences) supported by BD CellQuest Pro Software. We counted 10,000 cells/events and the results were expressed as mean intensity of fluorescence.

### Quantification of hydrogen peroxide (H_2_O_2_) production

The production of H_2_O_2_ was determined by using the procedure described by Pick and Keisari [28], adapted for microassay by Pick and Mizel [29]. This method is based on the oxidation of phenol red by H_2_O_2_, reaction peroxidase-dependent. MCF-7 cells (150 µL; 2.0×10^5^ cells per well) were cultured in 96-well microplates and incubated in medium containing 10% FBS. After incubation for 24 hours, cells were treated with 10 mM metformin solution and incubated for a further 24 hours at 37°C and 5% CO_2_. After this period, the cells were treated with catalase (20 µg mL^-1^) for 30 minutes. The medium was then removed, the microplate was washed with PBS and the cells incubated with the solution of phenol red (100 µL per well). Some cells were incubated in the presence of 10 µL of phorbol miristate acetate (an indirect activator of NADPH oxidase). The microplate was incubated at 37°C, for 1 hour. Finally, the reaction was blocked with 20 µL of sodium hydroxide solution (NaOH, 1 N) and the absorbance was determined by spectrophotometry at a wavelength of 620 nm. The absorbance was converted in nmoles of H_2_O_2_ using a standard curve of nmoles of H_2_O_2_ vs. absorbance. The treatment groups were compared with the control group and the results were expressed as nmoles of H_2_O_2_/∼8×10^5^ cells per hour.

### Trypan blue exclusion assay

MCF-7 cells (1 mL; 2.0×10^5^ cells per well) were cultured in 24-well microplates and incubated in medium containing 10% FBS. After incubation for 24 hours, cells were treated with culture medium (control), metformin solution (10 mM metformin), antioxidant enzymes (apocynin, superoxide dismutase (SOD), or catalase) or metformin + antioxidant enzymes (apocynin, SOD or catalase) and incubated for a further 48 hours at 37°C and 5% CO_2_. After this period, the medium was removed and the cells washed two times with PBS to remove the serum. Then, the number of viable cells and dead cells were counted using the Trypan blue dye solution which specifically stains dead cells. Trypan blue positive and negative cells were counted using a Neubauer chamber. Results were expressed in relation to total number of cells counted.

### Statistical analysis

Data are presented as the mean ±SEM. The data were analyzed using the Student's t-test for comparison of two groups, or by one-way analysis of variance (ANOVA) followed by Tukey's post-hoc test for multiple comparisons. Comparisons with p-values less than 0.05 were considered significant.

## Results

### Metformin induces cell cycle arrest in G_0_-G_1_ phase, increases cell apoptosis and cell necrosis in MCF-7 cells

**Figure 1:**
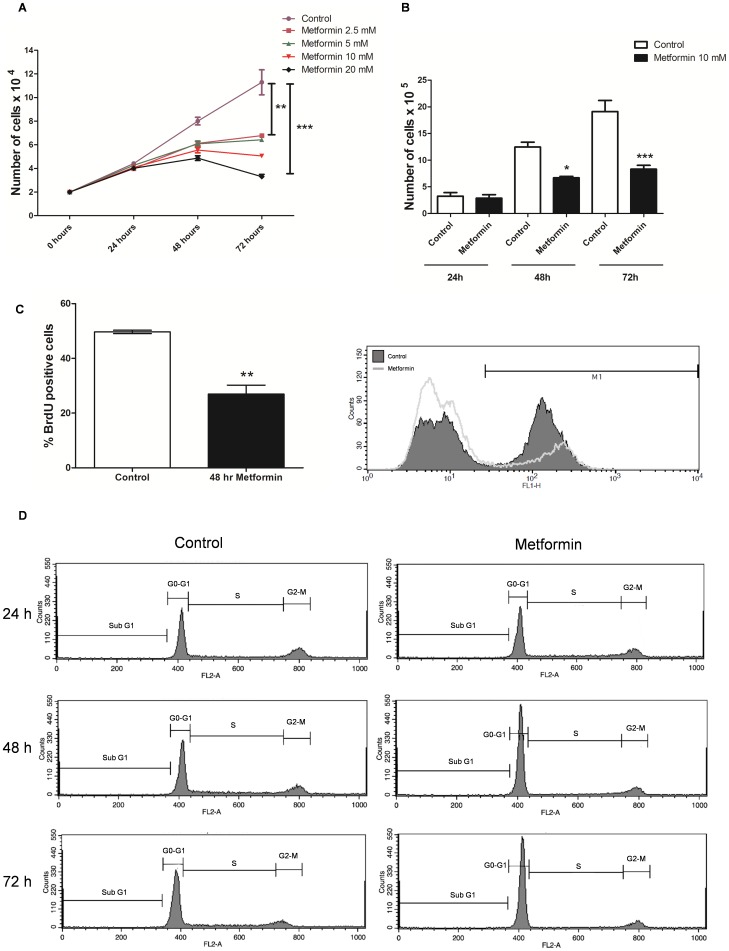
Metformin promoted antiproliferative activity in MCF-7 cells. (**A**) MCF-7 cells were treated with metformin (2.5, 5, 10, 20 mM) for 24, 48 and 72 hours and cell proliferation was analyzed using the MTT assay. Data was expressed as the number of viable cells compared with the control. (**B**) MCF-7 cells were treated with 10 mM metformin for 24, 48 and 72 hours and cell proliferation was analyzed by Trypan blue exclusion assay. Data was expressed as the number of viable cells compared with the control. (**C**) MCF-7 cells were treated with 10 mM metformin for 48 hours. Cell proliferation was determined by measuring BrdU incorporation using flow cytometry. (**D**) MCF-7 cells were treated with 10 mM metformin for 24, 48 and 72 hours. DNA cell cycle was analyzed by propidium iodide staining and measured by flow cytometry. Sub G1 phase, G_0_ – G_1_ phase, S phase, and G2-M phase were analyzed using flow cytometry. Results were reported in Table 1. * p<0.05 vs. control, ** p<0.001 vs. control, *** p<0.0001 vs. control.

Metformin demonstrated an antiproliferative activity in MCF-7 cells that was both time- and concentration-dependent. The difference in cell number was greater after 72 hours of metformin treatment compared with 24 and 48 hours (Figure 1 A) and this effect was greater with 20 mM of metformin as compared to 2.5, 5 and 10 mM (Figure 1 A). Furthermore, the number of viable cells analyzed by Trypan Blue exclusion assay in the metformin (10 mM for 48 and 72 hours) treated cells was lower than the control group further confirming its antiproliferative activity (Figure 1 B).

It was also found that MCF-7 cells treated with metformin had significantly less BrdU positive cells than the control indicating a decrease in cell proliferation and further supporting the antiproliferative nature of metformin treatment (Figure 1 C).

Treatment with metformin induced cell cycle arrest in the G_0_-G_1_ phase and increased cell apoptosis in a time-dependent manner. The percentage of cells in S phase decreased from ∼18% at 24 hours to ∼3% after 72 hours of metformin treatment. The proportion of cells in G_0_-G_1_ increased from ∼43% to ∼69% over this same interval. After 48 hours, the number of apoptotic cells (sub-G_1_ phase) was about 2-fold higher in the metformin group compared with the control group (Table 1; Figure 1 D).

**Table 1:** Metformin promoted cell cycle arrest and apoptosis.

		Sub G_1_	G_0_-G_1_	S	G2-M
**Control**	24	0.57±0.07	38.89±0.98	13.70±0.12	17.76±0.14
	48	0.49±0.10	47.55±0.48	20.45±0.41	15.14±0.30
	72	0.62±0.10	58.93±0.40	16.48±0.23	11.63±0.34
**Metformin**	24	0.77±0.04	42.53±0.35*	17.95±0.09***	15.05±0.21***
	48	0.84±0.05*	66.54±0.04***	8.15±0.26***	12.50±0.25**
	72	0.87±0.04	69.14±0.24***	3.38±0.19***	9.16±0.35**

MCF-7 cells were treated with metformin (10 mM for 24, 48 and 72 hours). DNA cell cycle was analyzed by propidium iodide staining and measured by flow cytometry. Values expressed in percent.

*p<0.05 vs. control;

**p<0.001 vs. control;

***p<0.0001 vs. control.

**Figure 2:**
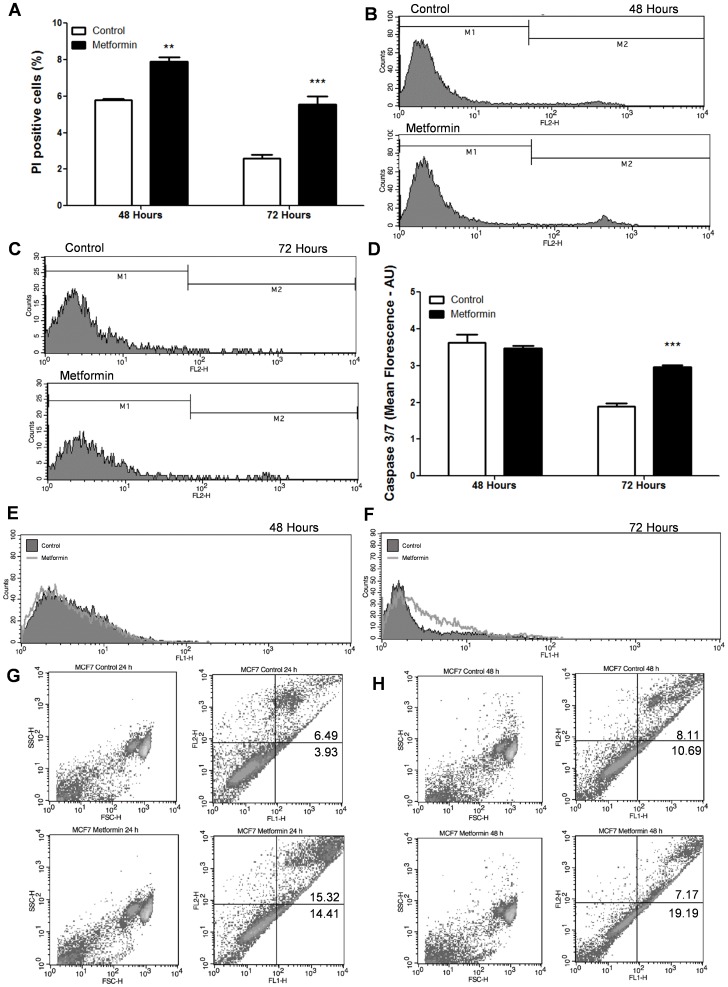
Metformin increased cell death and caspase activity in MCF-7 cells. (**A–C**) MCF-7 cells exposed to 10 mM metformin for 48 and 72 hours were analyzed with flow cytometry. Results were expressed as percentage of PI positive cells. (**D–F**) Apoptosis was determined by measuring active caspase-3 and -7 positive MCF-7 cells after 48 and 72 hours of treatment using flow cytometry. Results were expressed as mean fluorescence arbitrary units (AU). (**G–H**) MCF-7 cells were treated with 10 mM metformin for 24 and 48 hours. Apoptosis was determined by Annexin V propidium iodide staining and measured by flow cytometry. Apoptotic cells were measured as percentage of AV^+^/PI^-^ stained cells. ** p<0.001 vs. control, *** p<0.0001 vs. control.

Treatment with metformin (10 mM for 48 and 72 hours) increased cell necrosis as analyzed by PI staining and measured by flow cytometry (Figures 2 A–C). Metformin also increased the activity of caspases (3 and 7) after 72 hours of treatment as measured by flow cytometry. Active caspase-3 and -7 were significantly higher after 72 hours of treatment with metformin compared to the control (Figures 2 D-F). Similar results were observed in one set of samples that were analyzed for annexin V staining after 24 and 48 hours of metformin treatment (Figures 2 G and H).

### Metformin decreases the activation of insulin signaling pathways in MCF-7 cells

**Figure 3:**
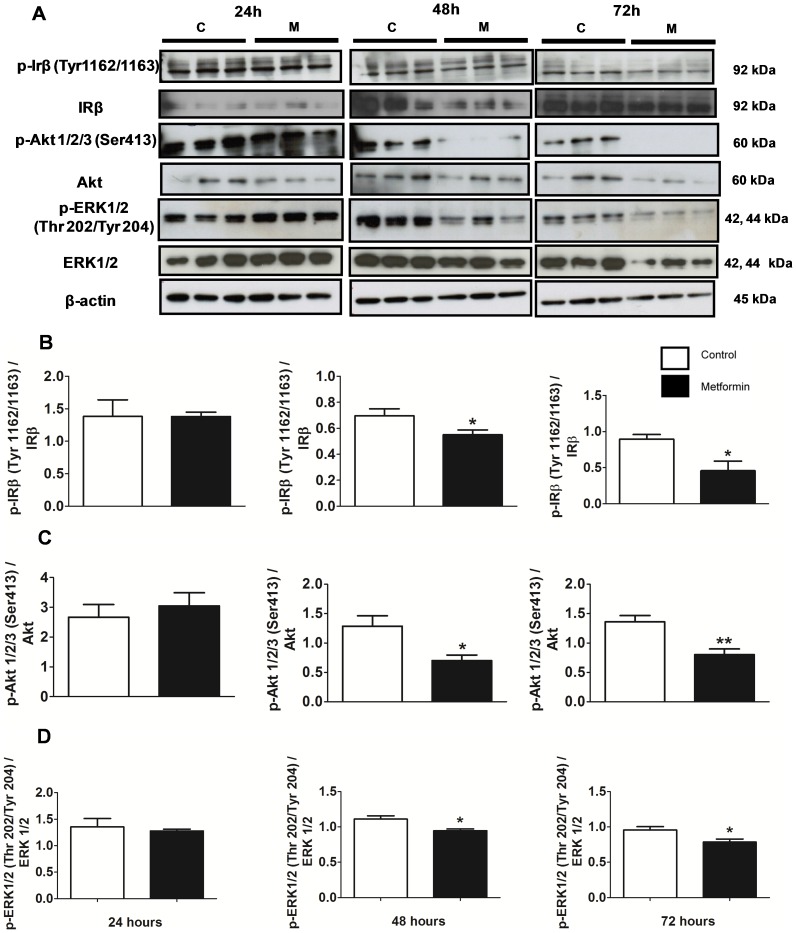
Metformin decreased the activation of insulin signaling pathway. (**A**) A western blot of IRβ, p-IRβ (Tyr 1162/1163), Akt, p-Akt1/2/3 (Ser 413), ERK1/2 and p-ERK1/2 (Thr 202/Tyr 204) with β-actin as the control band. (**B**) Western blot ratio analysis of p-IRβ (Tyr 1162/1163) and IRβ, (**C**) p-Akt1/2/3 (Ser 413) and Akt and (**D**) p-ERK1/2 (Thr 202/Tyr 204)and ERK1/2 in MCF-7 cells after treatment with metformin (10 mM) for 24, 48 and 72 hours. * p<0.05 vs. control; ** p<0.001 vs. control.

Treatment with metformin for 48 and 72 hours decreased the activation of the IR/PI3K/Akt/mTOR and IR/MEK/ERK1/2 pathways as demonstrated by a decrease in the expression of insulin receptor (IRβ), p-IRβ (Tyr1162/1163), Akt, p-Akt (Ser413) and p-ERK1/2 (Thr202/Tyr204) after 48 and 72 hours of treatment (Figures 3 A–D).

### Metformin is associated with AMPK and FOXO3a activation

**Figure 4:**
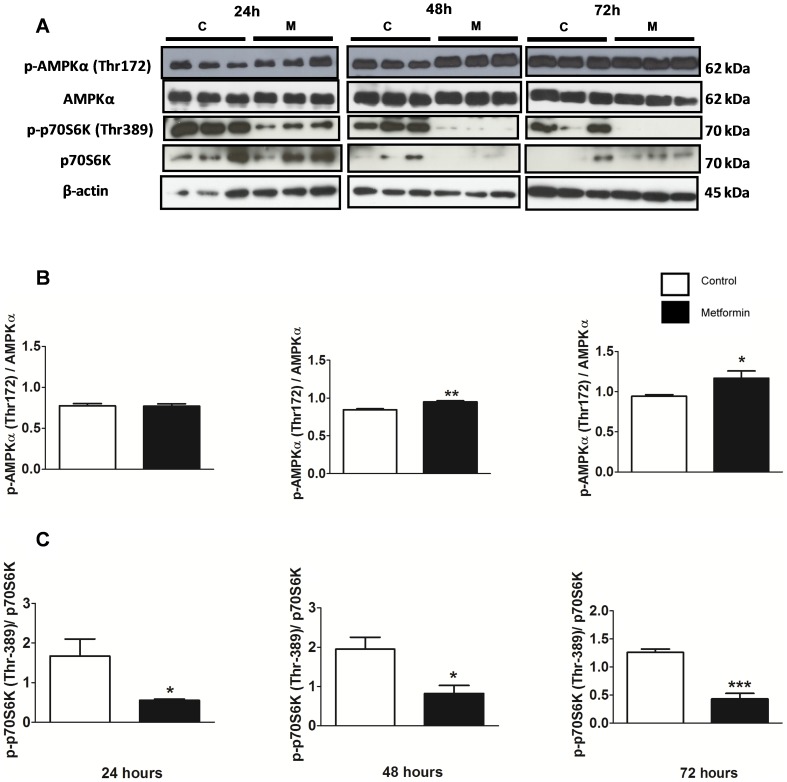
Metformin increased AMPK activity in MCF-7 cells. (**A**) A western blot of AMPKα, p-AMPKα (Thr 172), p70S6K and p-p70S6K (Thr 389) with β-actin as the control band. (**B**) Western blot ratio analysis of AMPK and p-AMPK (Thr 172), (**C**) p70S6K and p-p70S6K (Thr 389) in MCF-7 cells after treatment with metformin (10 mM) for 24, 48 and 72 hours. * p<0.05 vs. control; ** p<0.001 vs. control and *** p<0.0001 vs. control.

There was no significant difference in total AMPK expression between control and metformin treated groups. However, phosphorylation of Thr 172 within the alpha subunit was significantly increased in the metformin group compared with the control group after 48 and 72 hours of treatment (Figures 4 A and B). Furthermore, metformin decreased p70S6K phosphorylation at 24, 48 and 72 hours confirming the role of activated AMPK in MCF-7 cells treated with metformin and involvement of mTOR-p70S6K pathway in these cells (Figures 4 A and C).

**Figure 5:**
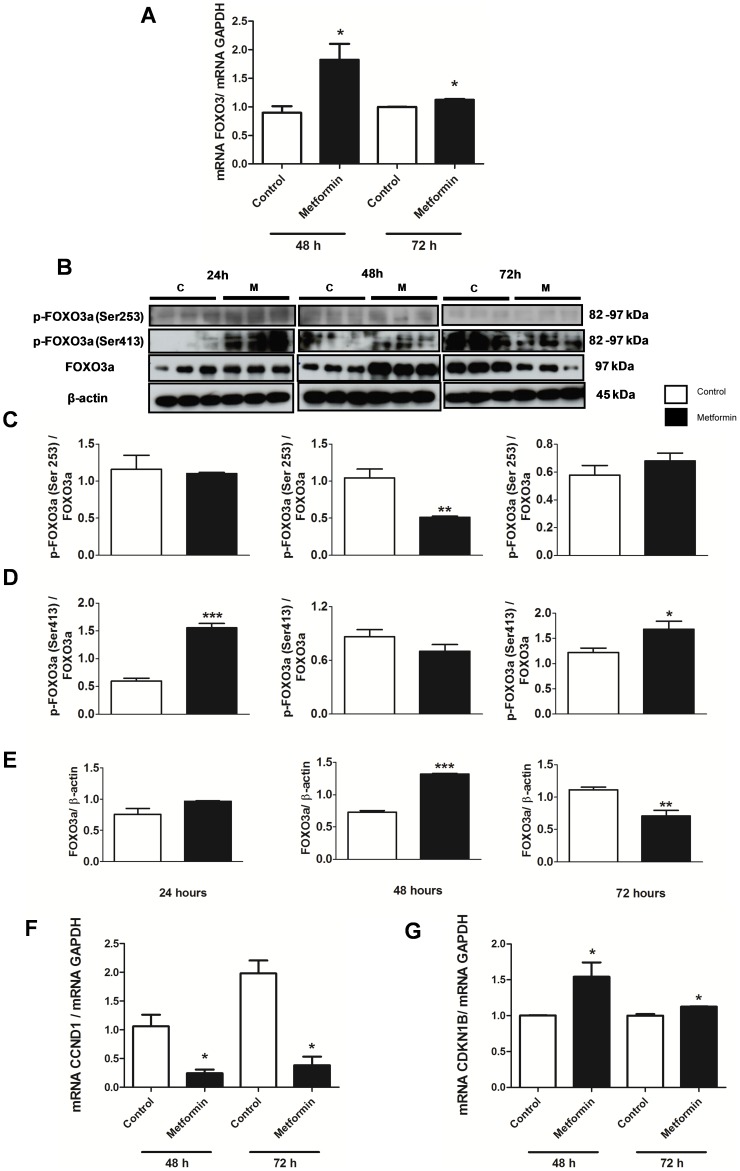
Metformin induced cell apoptosis associated with FOXO3a. (**A**) Real Time PCR ratio analysis of mRNA levels of FOXO3a after treatment with metformin (10 mM) for 48 and 72 hours in MCF-7 cells. (**B**) Western blot of p-FOXO3a (Ser 253), p-FOXO3a (Ser 413), FOXO3a and β-actin from MCF-7 cells after treatment with metformin (10 mM) for 24, 48 and 72 hours. (**C**) Western blot ratio analysis of p-FOXO3a (Ser 253) and FOXO3a, (**D**) p-FOXO3a (Ser 413) and FOXO3a, and (**E**) FOXO3a and β-actin. (**F**) Real Time PCR analysis of mRNA levels of cyclin D1 (CCND1 gene), and (**G**) p27 (CDKN1B gene), after treatment with metformin (10 mM) for 48 and 72 hours. * p<0.05 vs. control; ** p<0.001 vs. control and *** p<0.0001 vs. control.

FOXO3a mRNA expression was significantly higher after treatment with metformin for 48 and 72 hours when compared with the control group (Figure 5 A). The phosphorylation of FOXO3a at Ser 253 was significantly lower after 48 hours of metformin treatment when compared with the control, demonstrating an increase in FOXO3a activity mediated by the action of metformin (Figures 5 B and C). Furthermore, the phosphorylation of the FOXO3a at Ser 413, which is mediated by AMPK, was significantly higher after treatment with metformin for 24 and 72 hours when compared with control, demonstrating an increase in FOXO3a activity mediated by the action of metformin via AMPK activation (Figures 5 B and D). FOXO3a protein expression was significantly increased after treatment with metformin for 48 hours but decreased after 72 hours of treatment (Figures 5 B and E).

**Figure 6:**
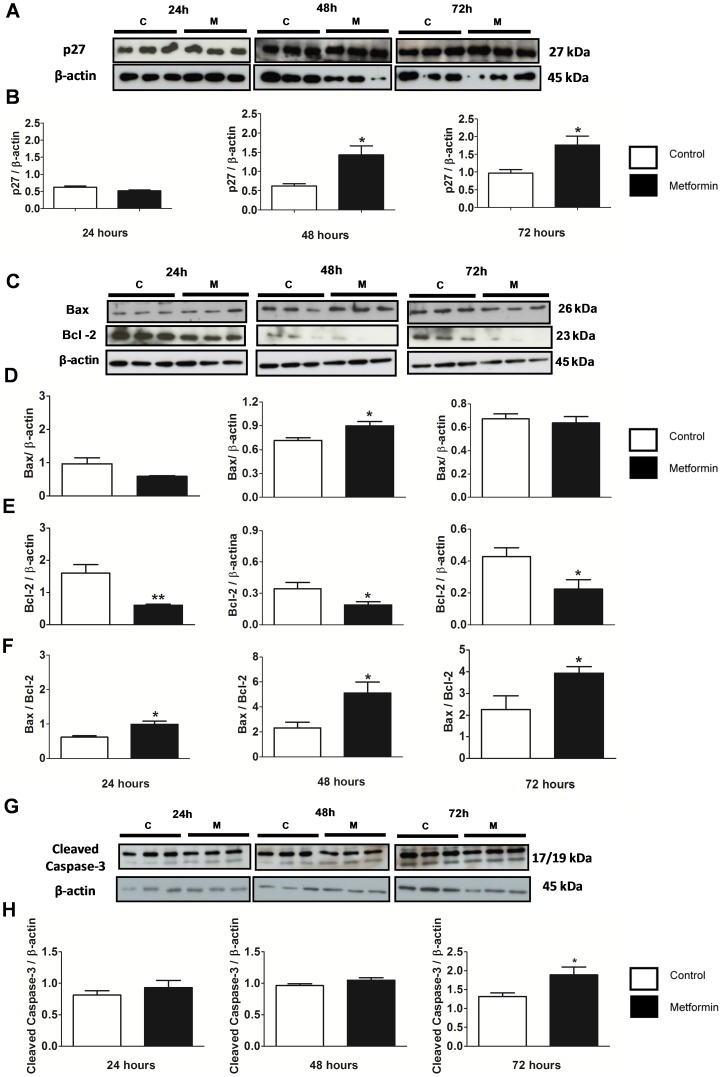
Metformin increased cell apoptosis as measured by p27, Bax, Bcl-2 and cleaved caspase-3 in MCF-7 cells. (**A**) Western blot of p27 and β-actin from MCF-7 cells that were treated with metformin (10 mM) for 24, 48 and 72 hours. (**B**) Western blot ratio analysis of p27 and β-actin. (**C**) Western blot of Bax, Bcl-2 and β-actin from MCF-7 cells that were treated with metformin (10 mM) for 24, 48 and 72 hours. (**D**) Western blot ratio analysis of Bax and β-actin, (**E**) Bcl-2 and β-actin, and (**F**) Bax and Bcl-2 at 24, 48 and 72 hours. (**G**) Western blot of cleaved caspase-3 and β-actin from MCF-7 cells that were treated with 10 mM metformin for 24, 48, and 72 hours. (**H**) Western blot ratio analysis of cleaved caspase-3 and β-actin at 24, 48, and 72 hours. * p<0.05 vs. control; ** p<0.001 vs. control.

Metformin was found to significantly decrease mRNA levels of cyclin D1 (CCND1) (Figure 5 F) and increase mRNA levels and protein expression of p27 (CDKN1B) after 48 and 72 hours of treatment (Figures 5 G and 6 A and B, respectively). Metformin increased mRNA levels of Bax and decreased that of Bcl-2 (data not shown). Furthermore, metformin increased the protein expression of Bax after 48 hours of treatment and decreased that of Bcl-2 after 24, 48 and 72 hours of treatment (Figures 6 C–E) and increased the Bax/Bcl-2 ratio (Figure 6 F), indicating the involvement of this pathway in cell apoptosis.

Metformin also increased the cleavage of caspase-3 (Figures 6 G and H) after 72 hours in accordance with the increase in activity of caspase-3 and 7 after 72 hours of treatment detected by flow cytometry (Figures 2 D–F).

### Antiproliferative effect of metformin is associated with oxidative stress

**Figure 7:**
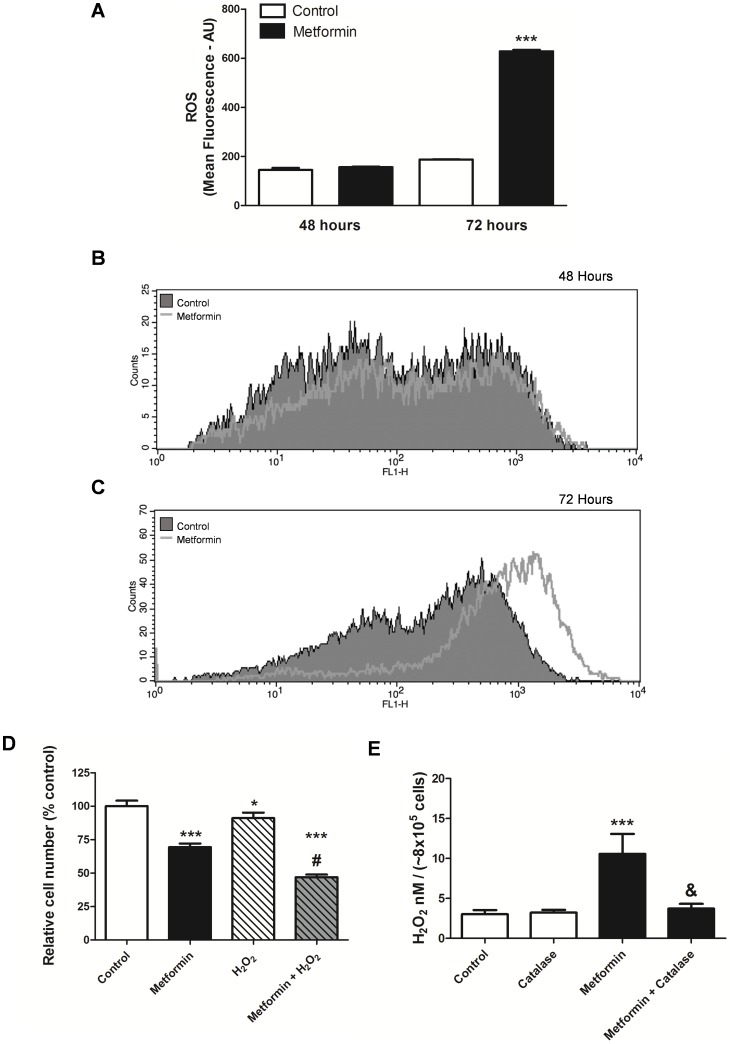
Antiproliferative effect of metformin was associated with oxidative stress and apoptosis. (**A–C**) MCF-7 cells were treated with metformin (10 mM) for 48 and 72 hours and ROS was determined by the CM-H_2_DCFDA assay, and measured by flow cytometry. (**D**) MCF-7 cells were treated with metformin (10 mM) for 48 hours, and then treated with H_2_O_2_, (1 mM) for an additional 4 hours and cell viability was analyzed. (**E**) Production of H_2_O_2_ by MCF-7 cells after treatment with metformin for 24 hours (10 mM) or metformin (10 mM) + catalase (20 µg mL^−1^). * p<0.05 vs. control; *** p<0.0001 vs. control; ^#^ p<0.001 vs. H_2_O_2_ and ^&^ p<0.0001 vs. metformin.

Treatment with metformin increased ROS production (Figure 7 A–C). ROS levels were 3-times higher in the metformin group compared with the control group after 72 hours of treatment (Figures 7 A and C).

To better understand the association of oxidative stress in relation to metformin, we analyzed the effect of co-treatment of MCF-7 cells with metformin and H_2_O_2_. The treatment of cells with 1 mM H_2_O_2_ reduced cell viability compared with the control, and the combined treatment of metformin and H_2_O_2_ further increased this effect at 48 hours (Figure 7 D). Metformin also increased the production of H_2_O_2_ after 24 hours and the treatment of metformin + catalase reduced it (Figure 7 E).

**Figure 8:**
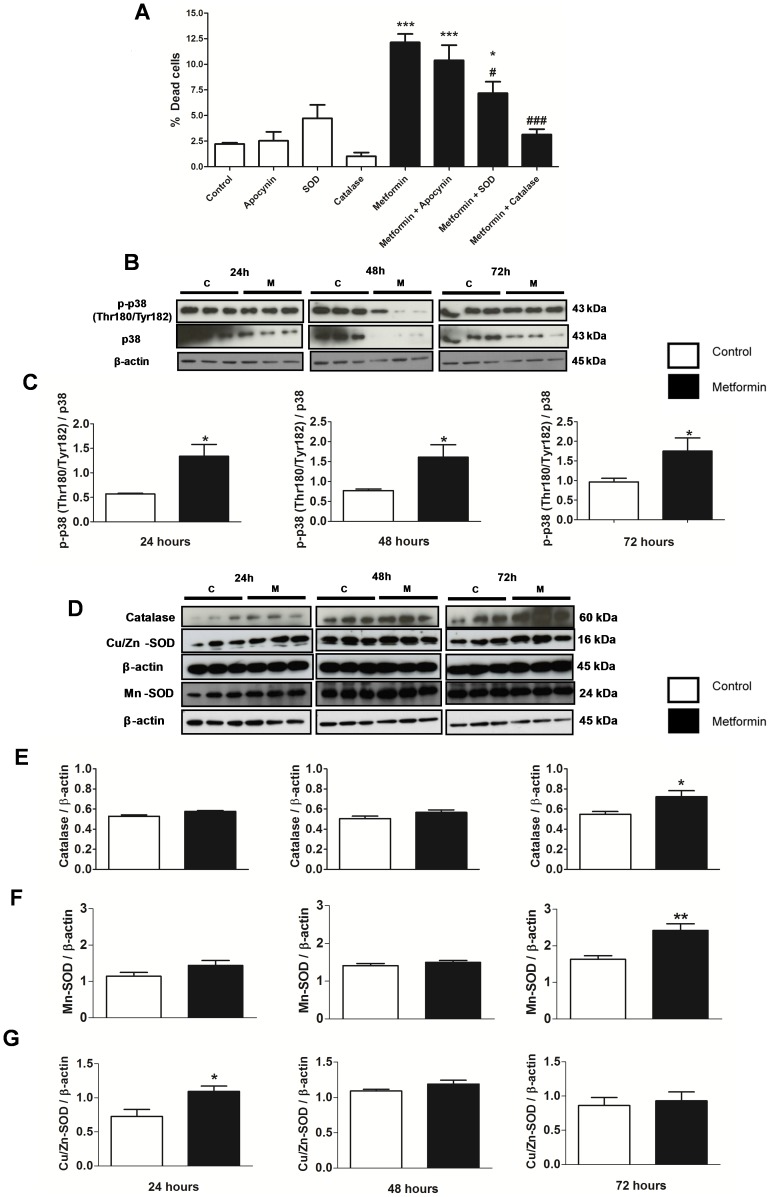
Antiproliferative effect of metformin was associated with oxidative stress and p-p38 MAPK activation. (**A**) MCF-7 cells were treated with metformin (10 mM) or metformin + antioxidant enzymes (apocynin, SOD or catalase) for 48 hours and percentage of dead cells was determined by Trypan blue exclusion assay. (**B–C**) Western blot and western blot ratio analysis of p38 and p-p38 MAPK (Thr 180/Tyr 182), and (**D–G**) Catalase, MnSOD and CuZnSOD in MCF-7 cells after treatment with metformin (10 mM) for 24, 48 and 72 hours. * p<0.05 vs. control; ** p<0.001 vs. control; *** p<0.0001 vs. control; ^#^ p<0.05 vs. metformin; and ^###^ p<0.001 vs. metformin.

To further explore the link between ROS formation and the antiproliferative effects of metformin, the effect of co-treatment of MCF-7 cells with metformin and antioxidant enzymes, apocynin, SOD and catalase was determined. The results of this experiment confirm the involvement, at least in part, of oxidative stress in the action of metformin, as the treatment with metformin for 48 hours increased the percentage of dead cells (Figure 8 A). The combined treatment of metformin and SOD or catalase reduced this metformin effect, since the percentage of dead cells was decreased (Figure 8 A). However, the percentage of dead cells with metformin + antioxidant enzymes was still higher than the control group, suggesting that other mechanisms are associated with the effect of metformin.

In accordance with the increase in ROS production after 72 hours of metformin treatment (Figure 7 A), we observed an increase in the p-p38 MAPK (Thr180/Tyr182), catalase and Mn-SOD protein expression (Figures 8 B–F) suggesting an adaptive antioxidant response to increased ROS load. However, Cu/Zn-SOD protein expression was increased only after 24 hours metformin treatment (Figure 8 G).

### Antiproliferative effect of metformin in LLC WRC-256 tumor cells

**Figure 9:**
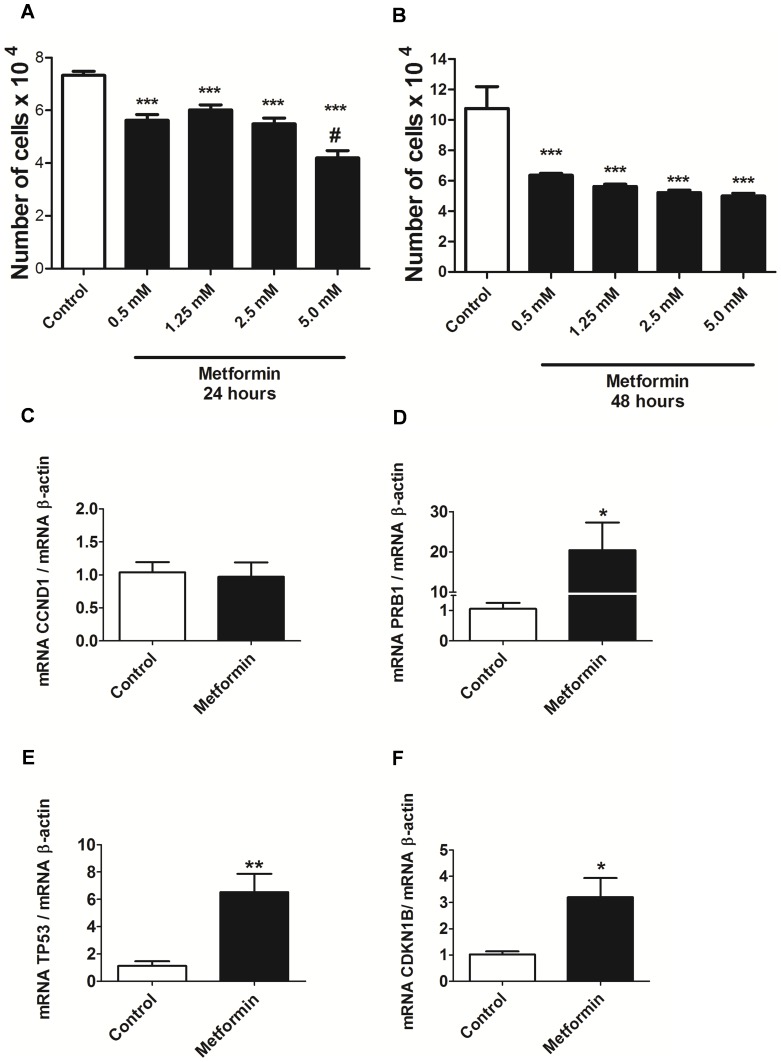
Metformin demonstrated antiproliferative effect in LLC WRC-256 tumor cells. LLC WRC-256 cells were treated with metformin (0.5, 1.25, 2.5 and 5 mM) for 24 (**A**) and 48 (**B**) hours and cell proliferation was analyzed using the MTT assay. Data was expressed as number of viable cells compared with the control. (**C–F**) mRNA levels of cyclin D1 (CCND1 gene), pRb (PRB1 gene), p53 (TP53 gene) and p27 (CDKN1B gene), respectively, after treatment with metformin (5 mM) for 48 hours in LLC-WRC-256 breast cancer cells using real time PCR. * p<0.05 vs Control, ** p<0.001 vs Control, *** p<0.0001 vs Control and ^#^ p<0.05 vs Metformin 2.5 mM.

Metformin demonstrated an antiproliferative activity in LLC WRC-256 tumor cells that was both time- and concentration-dependent. The antiproliferative activity was greater after 48 hours of treatment compared with 24 hours (Figures 9 A and B) and this effect was greater with 5 mM of metformin as compared to 0.5, 1.25 and 2.5 mM (Figure 9 A).

In addition, metformin significantly increased mRNA levels of pRb (PRB1 gene), p53 (TP53 gene) and p27 (CDKN1B gene) after 48 hours of treatment in LLC WRC-256 tumor cells (Figures 9 D–F, respectively). There was no difference in cyclin D1 mRNA (CCND1 gene) between control and metformin groups (Figure 9 C).

## Discussion

Recent studies have demonstrated that metformin exhibits antiproliferative effect in cancer cells both directly as well as indirectly by improving insulin sensitivity, and consequently decreasing hyperinsulinaemia [3], [9], [10]. The mechanism of action of metformin is well studied in the liver, adipose tissue, skeletal and heart muscles [7], [30]. Metformin can lower elevated insulin levels associated with type 2 diabetes by inhibiting hepatic gluconeogenesis via AMPK activation, and increases insulin sensitivity and glucose utilization by skeletal muscle and adipose tissue resulting in reduced blood glucose and insulin levels [7], [8], [30]–[32]. Insulin is an important growth factor for cancer cells and chronic hyperinsulinaemia has been associated with cancers of the colon, breast, pancreas, and endometrium [10], [33]–[35]. However, the mode of action and the biological consequences of metformin in cancer cells still remain poorly understood.

In the current study, we demonstrated that metformin inhibited proliferation of MCF-7 cells by promoting cell cycle arrest in the G_0_-G_1_ phase, inhibiting cyclin D1 and inducing cell apoptosis and necrosis. These effects were also associated with increased oxidative stress and the activation of AMPK and FOXO3a. A study by Sahra et al. [36], demonstrated that metformin was able to inhibit the proliferation of prostate cancer cells by blocking cell cycle in the G_0_-G_1_ phase and decreasing cell viability. However, in their study, metformin did not induce apoptosis. Other investigators have reported that metformin can increase apoptosis of breast, colon and endometrial cancer cells [14], [37], [38] using different mechanisms. Buzzai et al. [37] reported that apoptosis stimulated by metformin in colon cancer cells was associated with loss of p53-dependent enhancement of autophagy and glycolysis. Berstein et al. [14] and Zhuang and Miskimins [39] demonstrated that metformin alone, or in combination with tamoxifen, increased apoptosis in MCF-7 cells via caspase and poly-ADP-ribose polymerase (PARP). In the current study, we demonstrated a caspase mediated increase in cell apoptosis induced by metformin. These results indicate that an apoptotic mechanism is implicated in metformin induced inhibition of proliferation of MCF-7 cells.

Zhuang and Miskimins [39] also observed that PARP-dependent cell death was associated with accumulation of large and clear vacuoles representing enlargement of mitochondria. These alterations could be due to the effect of metformin in inhibiting complex I of the electron transport chain in mitochondria, as was suggested by Owen et al. [40]. In prostate cancer cells, it has been reported that metformin can lead to a loss of mitochondrial membrane potential and inhibition of ATP production that can consequently activate AMPK protein [34], [41]. Our results showed that treatment with metformin increased the expression of p-AMPK (Thr 172) and reduced the expression of p-p70S6K (Thr 389), which confirms the ability of metformin to activate AMPK and inactivate mTOR. Up-regulation of AMPK activity by metformin in MCF-7 cells has also been previously reported in the literature [15]. We have demonstrated that metformin not only activates AMPK and inactivates Akt, which are upstream of mTOR, but also inactivates p70S6K, which is downstream of this pathway, supporting the observation that AMPK/p70S6K pathway is a major target for metformin induced apoptosis in MCF-7 cells.

The PI3K/Akt/mTOR signaling pathway has an important role in cancer metabolism regulating proliferation and apoptosis. This pathway is downstream of several growth factor receptors such as insulin receptor and insulin like growth factor I receptor, which coordinate tumor growth [42], [43]. Insulin receptor activation can also stimulate the MEK/ERK1/2 MAPK (mitogen activated protein kinase) signaling pathway and contribute to tumor growth [44]–[47]. Our finding that metformin reduced insulin receptor activation in MCF-7 breast cancer cells represents an important activity of this drug in controlling cancer cell proliferation. Furthermore, we demonstrated a reduction in the activation of the ERK1/2 signaling pathways by metformin in MCF-7 cells which supports the antiproliferative effect of metformin. In an earlier study [9], we demonstrated that metformin, independent of improving insulin sensitivity, was effective in controlling tumor development in obese rats. The observed effect of metformin in decreasing tumor development may be due at least in part to its metabolic effects correcting lipid abnormalities, reducing accumulation of adipose tissues and keeping low circulating insulin levels. Furthermore, metformin bore a direct and effective action on the tumor breast cell, mainly by increasing the area of necrosis. The present study using MCF-7 cells further confirmed the role of metformin in increasing cell death.

Studies have shown that AMPK can activate FOXO proteins under certain conditions, such as nutrient deprivation, and this can promote tumor suppression [18], [19], [21]. FOXO plays a significant role in longevity and tumor suppression by up-regulating target genes involved in stress resistance, metabolism, cell cycle arrest and apoptosis. Environmental stimuli, such as insulin, nutrients, and oxidative stress control FOXO activity by regulating its protein levels, subcellular localization, DNA-binding properties, and transcriptional activity [20]. Overexpression of wild-type FOXO in *Caenorhabditis elegans* and *Drosophila melanogaster* has been reported to extend lifespan [48], [49]. In contrast, loss of FOXO expression has been associated with increased cancer in mammals [50], [51]. Cytoplasmic localization of FOXO3a has also been suggested to be correlated with poor survival in patients with breast cancer [52]. Our results demonstrate that metformin leads to FOXO3a activity in MCF-7 cells as there was an increase in mRNA level and protein expression for FOXO3a at 48 hours in the metformin-treated group. Furthermore, phosphorylation of FOXO3a at Ser 253 was significantly lower at 48 hours with metformin treatment, indicating an increase in its activity. The phosphorylation of FOXO3a by Akt at the Ser 253 residue may lead to an accumulation of FOXO3a in the cytoplasm and subsequent degradation [12], [20], [21]. In addition, the phosphorylation of FOXO3a at Ser 413 was significantly higher with metformin treatment indicating an increase in its activity. The phosphorylation of FOXO3a by AMPK at Ser 413 residue can increase FOXO3a activity independent of FOXO3a localization or DNA-binding activity. AMPK phosphorylation of FOXO3a induces changes in the expression of specific target genes including energy metabolism and stress resistance genes such as catalase and SOD [19]–[21], [53].

The significance of the antitumoral activity of FOXO3a is also highlighted in studies conducted on leukemia, prostate cancer and glioblastoma [54]–[56]. FOXO3a is required for colorectal cancer cell death induced by cisplatin and p38α inhibitors [18], [57]. Activated FOXO3a is able to bind to promoters and induce the transcription of target genes involved in cell cycle arrest and cell death contributing to tumor supression [12], [18], [21], [58].

An increase in p27 mRNA (CDKN1B gene) and p27 protein expression, and a decrease in cyclin D1 mRNA (CCND1 gene) level were observed in the metformin-treated group when compared to the control group. This suggests that cyclin D1 and p27, two important regulators of cell cycle, are intracellular targets of the metformin-mediated antiproliferative effect in MCF-7 cells via FOXO3a activity. The CCND1 gene is overexpressed in several types of human cancers [36], [59] and an increased expression of cyclin D1 in the prostate cancer cell line enhanced cell growth and tumorigenicity [60]. Therefore, decreasing cyclin D1 gene expression may be an important molecular target to control tumor proliferation. Recent studies have also demonstrated that metformin may specifically affect proliferation of cancer cells compared to normal cells [36], [39]. Zhuang and Miskimins [39] demonstrated that the non-transformed human mammary epithelial cell line, MCF10A, was resistant to the cytotoxic effects of metformin. In these cells, treatment with metformin (8 mM for 4 days) did not increase the amount of dead cells compared to control.

In addition, our results in LLC WRC-256 tumor cells confirm the antiproliferative effect of metformin in another cell line and the effect of metformin in the control of cell cycle, since an increase in pRb mRNA (PRB1 gene), p53 mRNA (TP53 gene) and p27 mRNA (CDKN1B gene) was also observed in this cell line.

The Bcl-2 family of proteins are important regulators of apoptosis. In mammals, Bcl-2 has at least twenty members, including four other anti-apoptotic proteins: Bcl-xL, Bcl-w, A1 and Mcl1, and two groups of proteins that promote cell death: Bax and BH3. Bax may homodimerize so that apoptosis is promoted, or it may heterodimerize with Bcl-2 and/or Bcl-xL, so that apoptosis is inhibited [61]–[63]. In our study, apoptosis induced by metformin in MCF-7 cells was associated with down-regulation of anti-apoptotic Bcl-2 expression and an increased Bax/Bcl-2 ratio. It has previously been demonstrated that the Bax/Bcl-2 ratio determines the susceptibility of cells to apoptosis [23], [64]. Bax and adenine nucleotide translocator cooperate within the permeability transition pore complex to increase mitochondrial membrane permeability. This increase in permeability results in the release of cytochrome *c*, apoptosis-inducing factor, and other pro-apoptotic molecules from the mitochondria into the cytoplasm. These apoptosis inducing molecules activate executioner caspases (3, 6 and 7) leading to apoptosis [65], [66]. Metformin treatment at 72 hours significantly increased active caspase-3/7 and cleaved-caspase-3 expression. It has previously been demonstrated that the executioner caspase-7 can activate downstream of mitochondria-mediated apoptosis in MCF-7 cells [67]. Therefore, the increase in caspase-3/7 activities observed in our study further supports the role of metformin in activating executioner caspases leading to apoptosis.

Cancer cells exhibit the Warburg effect, where even in the presence of oxygen, the cells prefer the much less efficient process of glucose fermentation (glycolysis) for energy production [68], [69]. This shift from aerobic energy production to anaerobic has received a lot of attention in terms of targeting cancer cell metabolism as a promising new strategy to treat cancer. Anastasiou et al. [70] showed that cancer cells can decrease the levels of ROS using this metabolic shift which can prevent oxidative damage. Therefore, alternatives to counteract oxidative stress seem to be a major prerequisite for cancer progression. Many of the agents that induce apoptosis are oxidants while many antioxidants may inhibit apoptosis [25]. Growth inhibition and ROS generation by metformin in MCF-7 cells indicate that ROS production is the cause of this apoptotic cell death. We have demonstrated that increased H_2_O_2_ production with metformin in MCF-7 cells was associated with increased antiproliferative effect. However, the combination of metformin with antioxidant enzymes, SOD and catalase, decreased the antiproliferative effect by decreasing the percentage of dead cells. These data suggest that the metformin effect in MCF-7 cells is partly mediated by oxidative stress.

FOXO proteins, in response to oxidative stress, induce cell cycle arrest, repair damaged DNA or induce apoptosis by modulating genes that control these processes [12], [21], [51]. It is also known that in mammalian cells, Akt, ERK 1/2, p38 MAPK and JNK [71], [72] regulate mitosis, metabolism and cellular apoptosis [71]–[75]. Specifically, Akt and ERKs are usually activated by growth signals, such as insulin and nutrients, while p38 MAPK and JNK are often activated by stress-responsive signals [71]. Activation of Akt and ERK leads to phosphorylation and inactivation of FOXO proteins, whereas p38 activation leads to FOXO3a activation. Our results demonstrate that oxidative stress, AMPK and p38 activation, as well as Akt and ERK 1/2 inactivation by metformin contributed to FOXO3a activation leading to cell cycle arrest and apoptosis of MCF-7 cells. Our study has suggested that metformin may represent an important therapeutic agent in the inhibition of breast tumor growth.

## Conclusions

**Figure 10:**
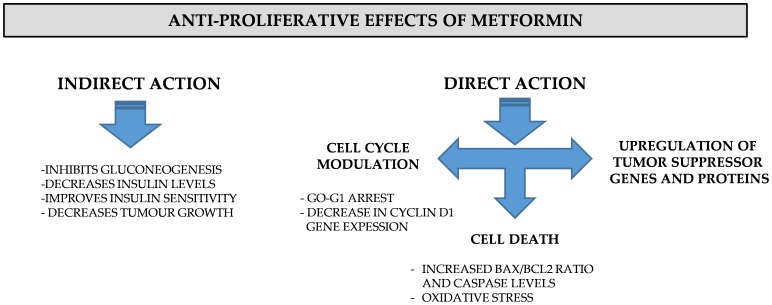
The direct and indirect antiproliferative effects of metformin. Schematic representation of the effects of metformin on tumor development. The indirect mechanism of action of metformin is mediated by the improvement of insulin sensitivity and decreased insulin levels which consequently decreases tumor growth. The direct mechanism of action of metformin is associated with cell cycle modulation, cell death and up-regulation of tumor suppression genes.

In the current study, we have demonstrated that metformin inhibits the proliferation of MCF-7 cells as a consequence of promoting cell cycle arrest in the G_0_-G_1_ phase, cell apoptosis and cell death. These effects were associated with oxidative stress, AMPK and FOXO3a activation. Our study further reinforces the potential benefit of metformin in cancer treatment, and provides detailed mechanistic insight into the antiproliferative effect of metformin through activation of FOXO3a transcription factor in breast cancer MCF-7 cells. Based on our own present findings and the data available in the literature, it is evident that metformin can indirectly exhibit antiproliferative effects as well as directly inhibit cell proliferation via cell cycle modulation, up regulation of tumor suppressor genes and by inducing cell death mediated by increased oxidative stress (Figure 10).
